# IFNAR2 relevance in the clinical outcome of individuals with severe COVID-19

**DOI:** 10.3389/fimmu.2022.949413

**Published:** 2022-07-29

**Authors:** Ingrid Fricke-Galindo, Alfonso Martínez-Morales, Leslie Chávez-Galán, Ranferi Ocaña-Guzmán, Ivette Buendía-Roldán, Gloria Pérez-Rubio, Rafael de Jesus Hernández-Zenteno, Abigail Verónica-Aguilar, Aimé Alarcón-Dionet, Hiram Aguilar-Duran, Ilse Adriana Gutiérrez-Pérez, Oscar Zaragoza-García, Jesús Alanis-Ponce, Angel Camarena, Brandon Bautista-Becerril, Karol J. Nava-Quiroz, Mayra Mejía, Iris Paola Guzmán-Guzmán, Ramcés Falfán-Valencia

**Affiliations:** ^1^ HLA Laboratory, Instituto Nacional de Enfermedades Respiratorias Ismael Cosío Villegas, Mexico City, Mexico; ^2^ Laboratory of Integrative Immunology, Instituto Nacional de Enfermedades Respiratorias Ismael Cosío Villegas, Mexico City, Mexico; ^3^ Translational Research Laboratory on Aging and Pulmonary Fibrosis, Instituto Nacional de Enfermedades Respiratorias Ismael Cosío Villegas, Mexico City, Mexico; ^4^ COPD Clinic, Instituto Nacional de Enfermedades Respiratorias Ismael Cosío Villegas, Mexico City, Mexico; ^5^ Faculty of Chemical-Biological Sciences, Universidad Autónoma de Guerrero, Chilpancingo, Mexico; ^6^ Interstitial Pulmonary Diseases and Rheumatology Unit, Instituto Nacional de Enfermedades Respiratorias Ismael Cosio Villegas, Mexico City, Mexico

**Keywords:** IFNAR2, COVID-19, genetic susceptibility, interferon alpha-beta, innate immunity, SNP, severe COVID-19, mortality risk

## Abstract

Interferons (IFNs) are a group of cytokines with antiviral, antiproliferative, antiangiogenic, and immunomodulatory activities. Type I IFNs amplify and propagate the antiviral response by interacting with their receptors, IFNAR1 and IFNAR2. In COVID-19, the *IFNAR2* (interferon alpha and beta receptor subunit 2) gene has been associated with the severity of the disease, but the soluble receptor (sIFNAR2) levels have not been investigated. We aimed to evaluate the association of *IFNAR2* variants (rs2236757, rs1051393, rs3153, rs2834158, and rs2229207) with COVID-19 mortality and to assess if there was a relation between the genetic variants and/or the clinical outcome, with the levels of sIFNAR2 in plasma samples from hospitalized individuals with severe COVID-19. We included 1,202 subjects with severe COVID-19. The genetic variants were determined by employing Taqman^®^ assays. The levels of sIFNAR2 were determined with ELISA in plasma samples from a subgroup of 351 individuals. The rs2236757, rs3153, rs1051393, and rs2834158 variants were associated with mortality risk among patients with severe COVID-19. Higher levels of sIFNAR2 were observed in survivors of COVID-19 compared to the group of non-survivors, which was not related to the studied *IFNAR2* genetic variants. IFNAR2, both gene, and soluble protein, are relevant in the clinical outcome of patients hospitalized with severe COVID-19.

## Introduction

Interferons (IFNs) are a group of pleiotropic cytokines based upon the expression of thousands of interferon-stimulated genes (ISGs), such as antiviral, antiproliferative, antiangiogenic, and immunomodulatory activities (1). The innate IFN type I and III (α/β and γ, respectively) amplify and propagate the antiviral response. While responses to IFN-λ are limited by receptor expression to the mucosal epithelium, all nucleated cells respond to IFN-α/β, being this IFN essential in the antiviral defense mechanism (2).

Type I IFN binds to the receptor complex composed of IFN-α/β receptors 1 and 2 (IFNAR1 and IFNAR2, respectively), associated with the Janus kinases, Tyk2 and Jak1, respectively. The activation of these kinases produces the tyrosine phosphorylation of STAT1 and STAT2, leading to the formation of a heterotrimer with the IFN-stimulated gene factor 3 (ISGF3) transcription factor and with the IRF-family member IRF-9 (1). The IFNAR2 subunit has a soluble isoform (sIFNAR2) that can be produced by alternative splicing of the *IFNAR2* (interferon alpha and beta receptor subunit 2) gene through a transcript that lacks the transmembrane and cytoplasmic domain (3) or can be cleaved by specific proteases such as TNF-alpha converting enzyme (known as TACE or ADAMS) and presenilins (PSEN) (4).

There are scarce studies of sIFNAR2 levels in body fluids. However, differences in the levels of this receptor have been reported in patients with multiple sclerosis (5), in variable clinical response to IFN-β treatment in the same disorder (6), as well as in cytomegalovirus-related vascular pathologies (7). Likewise, investigations including genetic variants in *IFNAR2* are limited, but rare mutations in this gene have been found in patients with immunodeficiency after measles-mumps-rubella vaccination (2, 8).

In coronavirus disease 2019 (COVID-19), *IFNAR2* has demonstrated relevance in the available genetic association studies. Pairo-Castineira, in collaboration with different consortiums, performed a GenOMICC (Genetics Of Mortality In Critical Care) genome-wide association study in 2,244 critically ill patients with COVID-19 from 208 UK intensive care units. They reported that the rs2236757 *IFNAR2* variant is associated with critical illness among individuals with COVID-19 (9). The *locus* also showed pleiotropic association with COVID-19 severity using the summary data-based Mendelian randomization (SMR) method (10). Likewise, other studies using different methodologies have identified *IFNAR2* as an important causal gene of COVID-19 severity (11–14), although the levels of the soluble receptor have not been determined.

Single-nucleotide variants (SNVs) in *IFNAR2* could lead to variation in the receptor structure, affect the binding site to IFN, or alter the gene expression (15). Currently, *IFNAR2* SNVs have not been widely studied, but several of them have been investigated in the susceptibility to hepatitis B virus (16), and an utterly IFNAR2 deficiency was observed in cases of encephalitis-induced following measles, mumps, and rubella vaccination (2).

We aimed to evaluate the association of *IFNAR2* SNVs (rs2236757, rs1051393, rs3153, rs2834158, and rs2229207) with COVID-19 mortality and to assess if there was a relation between the genetic variants and/or the clinical outcome, with the levels of sIFNAR2 in plasma samples from hospitalized subjects with severe COVID-19.

## Subjects and methods

### Subjects

We included 1,202 individuals with COVID-19, hospitalized in the Instituto Nacional de Enfermedades Respiratorias Ismael Cosio Villegas (Mexico City, Mexico) from July 2020 to March 2021. All patients were ≥18 years old and had a SARS-CoV-2 infection confirmed by reverse transcriptase-polymerase chain reaction (RT-PCR) test. The study protocol was approved by the local Research Ethics Committee (C53-20) and complied with the Helsinki Declaration statements. Each participant or patient’s relative was informed about the study and signed informed consent before the sample acquisition.

The patients enrolled presented a severe COVID-19 since they had dyspnea, a respiratory rate ≥30 breaths per minute, blood oxygen saturation ≤90%, and PaO_2_/FiO_2_ ≤300. The clinical outcome evaluated was the in-hospital mortality; subjects were classified as survivors if they were discharged from the hospital once a clinical improvement was achieved and non-survivors if they died during the hospital stay.

### Genotyping

Genomic DNA was isolated by standard techniques from a blood sample collected in tubes with EDTA as an anticoagulant. The *IFNAR2* rs2236757, rs1051393, rs3153, rs2834158, and rs2229207 were assessed by Taqman^®^ assays (C__11354003_30, C___2443247_30, C___9479908_10, C__16072683_20, C__16172148_10), according to the supplier instructions, employing a StepOnePlus™ Real-Time PCR System (Applied Biosystems, Carlsbad, CA, USA). The *IFNAR2* SNVs were selected according to a review of the scientific literature, the minor allele frequencies of the variants in Mexican, American, or Latin American populations, and the availability of genotyping methodologies. Hardy-Weinberg equilibrium and linkage disequilibrium analyses were assessed in Haploview (17).

### Determination of soluble IFNAR2 levels in plasma samples

The determination of the sIFNAR2 was performed in a subgroup of 351 individuals, chosen from a total of 1,202 according to the following criteria: a) *IFNAR2* genotypes, b) the clinical outcome, and c) the sampling time considering the days since symptoms onset. The sIFNAR2 levels were measured in plasma samples acquired between 0 and 15 days after the onset of the symptoms. The plasma samples were obtained by centrifugation of blood samples in EDTA tubes at 4500 rpm for 5 minutes and stored at -80°C until assayed. The soluble form of the subunit receptor was determined by the Human IFN alpha/beta R2 ELISA Kit of Invitrogen (Catalog # EH248RB, Life Technologies Corporation, Carlsbad, CA, USA), following the manufacturer’s protocol. A standard curve was generated for each plate including the following concentrations: blank, 0.16 ng/mL, 0.41 ng/mL, 1.02 ng/mL, 2.56 ng/mL, 6.4 ng/mL, and 16 ng/mL. The absorbance was read at 450 nm. Data were processed using computer software that plots the mean absorbance (y-axis) against the protein concentration (x-axis). The supplier’s recommended reduction method was employed to interpolate the samples’ absorbance for the concentration estimation. All samples were assessed by duplicate, reporting in ng/mL the mean values of the wells.

The blood group was determined by the serological test with a Novaclone^®^ kit (Licon, Mexico City, Mexico) to assess the influence of the blood groups on the sIFNAR2 plasma levels. For this analysis, blood group data was only available for 302 individuals.

### Statistical analyses

Continuous data are presented as the median and interquartile range (IQR), and categorical data are as frequencies in percentage. Normal distribution was assessed employing the Kolmogorov-Smirnov test. The association study of *IFNAR2* variants was performed in PLINK v1.07 (18). As required, the sIFNAR2 values were compared with Mann-Whitney U, Kruskal-Wallis, or Spearman’s rank correlation tests. The results were evaluated for multiple comparisons with the Benjamini-Hochberg method. The statistical analysis was performed in R/Rstudio (19).

## Results

### Clinical and demographic data of individuals with severe COVID-19

Four-hundred and twenty-six (35.4%) individuals with severe COVID-19 died during their hospital stay. Non-survivors were older (63 vs. 56 years old) and more frequently male than survivors (OR=1.36, CI 95%=1.05-1.75). Comorbidities were more frequent among non-survivors, but we observed significant differences for pre-existing chronic respiratory (OR=1.66, CI 95%=1.10-2.52) and ischemic heart (OR=2.33, CI 95%=1.30-4.20) diseases. A tendency was observed for systemic arterial hypertension (p=0.06). Meanwhile, most individuals in the non-survivor group required invasive mechanical ventilation (IMV), and their hospital stay was longer for this group.

Dyspnea, cough, and fever were the most common symptoms reported for individuals with severe COVID-19 in both groups, while anosmia and emesis were the least frequent clinical manifestations. We observed significant differences in fever, myalgia, ageusia, chest pain, and anosmia, and these symptoms were more frequent among the survivors’ group than among non-survivors (**Table 1**).

**Table 1:** Demographic and clinical data of patients with severe COVID-19.

	Non-survivors, n = 426 (%)^a^	Survivors, n = 776 (%)^a^	p^b^
**Age, years**	63 (55-71)	56 (48-64)	<0.001
**Sex (n,%)** Male Female	301 (70.7) 125 (29.3)	496 (63.9) 280 (36.1)	0.018
**Smoking**	128 (30.0)	217 (28.0)	0.460
**T2DM**	134 (31.5)	203 (26.3)	0.060
**Pre-existing Respiratory disease**	46 (10.8)	53 (6.9)	0.020
**Ischemic heart disease**	26 (6.1)	21 (2.7)	0.005
**SAH**	162 (38.2)	254 (32.7)	0.060
**IMV**	395 (92.7)	468 (60.3)	<0.001
**Length IMV, days**	18.8 (11-28)	7 (0-16)	<0.001
**BMI, kg/m^2^**	28.74 (25.7-33.1)	29.7 (26.6-33.3)	0.025
**Symptoms onset, days**	8 (4-8)	8 (5-9)	0.122
**Hospital stay, days**	20 (13-29)	18 (11-28)	0.020
**Symptoms (n,%)**			
** Dyspnea**	361 (84.7)	651 (84.2)	0.860
** Cough**	289 (68.0)	526 (68.0)	1.000
** Fever**	285 (67.1)	575 (74.3)	0.010
** Myalgia**	253 (59.5)	511 (66.1)	0.020
** Arthralgia**	248 (58.2)	485 (62.7)	0.130
** Headache**	183 (43.1)	353 (45.6)	0.420
** Odynophagia**	97 (22.8)	203 (26.3)	0.180
** Rhinorrhea**	73 (17.1)	118 (15.3)	0.410
** Ageusia**	41 (9.6)	116 (15.0)	0.010
** Diarrhea**	38 (8.9)	82 (10.6)	0.360^a^
** Chest pain**	31 (7.3)	88 (11.4)	0.026
** Anosmia**	14 (3.3)	68 (8.8)	<0.001
** Emesis**	11 (2.6)	25 (3.2)	0.730

Continuous data are presented as median (interquartile range, IQR) and categorical data as n and frequency in percentage (%). ^a^Clinical data were not available for some individuals. ^b^Statistical tests employed for the comparisons: Mann-Whitney U and Fisher’s Exact Test. BMI, body mass index; IMV, invasive mechanical ventilation; SAH, systemic arterial hypertension; T2DM, Type 2 diabetes mellitus.

### *IFNAR2* single-nucleotide variants are associated with clinical outcomes among individuals with severe COVID-19

The allele and genotype frequencies of *IFNAR2* SNVs are presented in **Table 2**. The genotypic frequencies of *IFNAR2* single-nucleotide variants accomplish with Hardy-Weinberg equilibrium, except for the rs2236757. The minor alleles of the rs2834158, rs3153, and rs1051393 were more frequent in the non-survivor group than in survivors. For the rs2229207 variant, there were no significant differences in the allele and genetic frequencies among the studied groups.

**Table 2:** Genetic association study of *IFNAR2* variants with mortality in patients with severe COVID-19.

*IFNAR2* single-nucleotide variant		All, n = 1202	Non-survivors, n = 426	Survivors, n = 776	p	OR (CI 95%)	FDR^b^
**rs2834158**							
**TT** **TC** **CC**		412 (0.343) 563 (0.468) 227 (0.189)	126 (0.296) 209 (0.490) 91 (0.214)	286 (0.368) 354 (0.456) 136 (0.175)	0.029	1 (reference) 1.34 (1.02-1.75) 1.51 (1.08-2.13)	0.072
**T** **C**		1,387 (0.577) 1,017 (0.423)	461 (0.541) 391 (0.459)	926 (0.597) 626 (0.403)	0.008	1.25 (1.06-1.48)	0.029
**rs2236757^a^**							
**AA** **AG** **GG**		396 (0.330) 541 (0.450) 265 (0.220	119 (0.280) 205 (0.481) 102 (0.239)	277 (0.356) 336 (0.433) 163 (0.210)	0.023	1 (reference) 1.42 (1.07-1.87) 1.45 (1.04-2.02)	0.116
**A** **G**		1,333 (0.554) 1,071 (0.446)	443 (0.520) 409 (0.480)	890 (0.573) 662 (0.427)	0.012	1.24 (1.05-1.47)	0.029
**rs3153**							
**AA** **AG** **GG**		400 (0.333) 564 (0.469) 238 (0.198)	122 (0.286) 212 (0.498)) 92 (0.216)	278 (0.358) 352 (0.454) 146 (0.188)	0.039	1 (reference) 1.37 (1.04-1.80) 1.43 (1.02-2.01)	0.065
**A** **G**		1,364 (0.567) 1,040 (0.433)	456 (0.535) 396 (0.465)	908 (0.585) 644 (0.415)	0.018	1.22 (1.03-1.45)	0.030
**rs1051393**							
**GG** **GT** **TT**	389 (0.324) 578 (0.481) 235 (0.195)		122 (0.286) 212 (0.498) 92 (0.216)	267 (0.344) 366 (0.472) 143 (0.184)	0.099	NA	0.124
**G** **T**	1,356 (0.564) 1,048 (0.436)		456(0.535) 396 (0.465)	900 (0.580) 652 (0.420)	0.035	1.20 (1.01-1.42)	0.043
**rs2229207**							
**TT** **TC** **CC**	811 (0.675) 348 (0.289) 43 (0.036)		286 (0.671) 125 (0.293) 15 (0.035)	525 (0.677) 223 (0.287) 28 (0.036)	0.974	NA	0.974
**T** **C**	1,970 (0.819) 434 (0.181)		697 (0.818) 155 (0.182)	1,273 (0.820) 279 (0.180)	0.895	NA	0.895

^a^Deviation from Hardy-Weinberg Equilibrium p<0.01; ^b^Benjamini-Hochberg method. CI, confidence interval; FDR, false discovery rate; NA, does not apply; OR, odds ratio.

The genotype frequencies of rs2834158, rs2236757, and rs3153 differed between the studied groups, although the statistical significance did not remain after correction for multiple comparisons. However, these two same variants were associated with mortality risk in the analysis of the dominant model (**Table 3**). Regarding the recessive model, there were no significant differences in the genotype frequencies between the study groups (**Supplementary Table 1**).

**Table 3:** Dominant model analyses for *IFNAR2* genetic variants were included in the study.

*IFNAR2* single-nucleotide variant	Genotypes	Non-survivors n = 426	Survivors n = 776	p	OR (CI 95%)	FDR^b^
**rs2834158**	TT TC + CC	126 (0.296) 300 (0.704)	286 (0.369) 490 (0.631)	0.011	1.38 (1.07-1.79)	0.027
**rs2236757^a^**	AA AG +GG	119 (0.279) 307 (0.721)	277 (0.357) 499 (0.643)	0.006	1.43 (1.10-1.85)	0.030
**rs3153**	AA AG + GG	122 (0.286) 304 (0.714)	278 (0.358) 498 (0.642)	0.011	1.39 (1.07-1.79)	0.019
**rs1051393**	GG GT + TT	122 (0.286) 304 (0.714)	267 (0.344) 509 (0.656)	0.041	1.3 (1.01-1.69)	0.061
**rs2229207**	TT TC + CC	286 (0.671) 140 (0.329)	525 (0.677) 251 (0.323)	0.854	NA	0.854

^a^Deviation from Hardy Weinberg Equilibrium p<0.01; ^b^Benjamini-Hochberg method. CI, confidence interval; FDR, false discovery rate; NA, it does not apply; OR, odds ratio.

In addition, we performed a linkage disequilibrium (LD) analysis for the *IFNAR2* variants included in the study. High *D’* values (*D’*>0.80) were observed for the four variants included in the analysis (the rs2236757 was excluded due to deviation to Hardy-Weinberg equilibrium) (**Supplementary Figure 1a**); however, a low *r^2^* was observed for the rs2229207 with the rs3153, rs1051393, and rs2834158 (*r^2^ = *0.12) (**Supplementary Figure 1b**). The solid spine method formed one block including the four variants (rs3153/rs2229207/rs1051393/rs2834158). According to the allele combinations, the haplotypes ATGT and GTGC were associated with low and high mortality risk, respectively (**Table 4**).

**Table 4:** Association analysis of *IFNAR2* haplotypes (rs3153/rs2229207/rs1051393/rs2834158) with mortality risk among patients with severe COVID-19.

Haplotypes	Frequencies		p	OR
	Non-survivors n = 426	Survivors n = 776		
**GTTC**	0.421	0.383	0.068	NA
**ATGT**	0.334	0.391	0.005	0.78 (0.65-0.93)
**ACGT**	0.166	0.161	0.740	NA
**ATTT**	0.025	0.024	0.840	NA
**GTGC**	0.020	0.010	0.040	2.08 (1.03-4.19)
**GTGT**	0.010	0.013	0.600	NA

Linkage disequilibrium analysis performed in Haploview, Block studied through the solid spine method. OR, odds ratio; NA, it does not apply.

### The levels of soluble IFNAR2 are related to the clinical outcome of COVID-19

**Figure 1:**
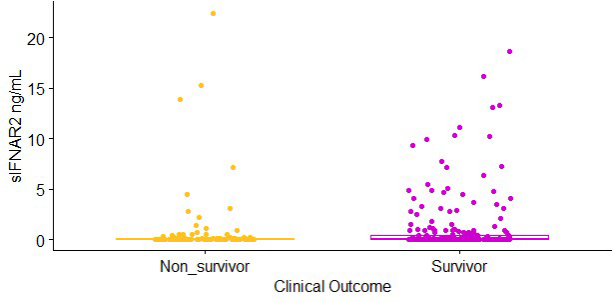
Soluble IFNAR2 (sIFNAR2) plasma levels of severe COVID-19 patients (n = 347) divided into non-survivor (n = 108, yellow dots) and survivor (n = 239, purple dots). sIFNAR2 level was evaluated by ELISA. Statistical comparison was performed using Mann-Whitney U Test, p < 0.05.

The sIFNAR2 levels were determined in 351 subjects with severe COVID-19. Low values of sIFNAR2 (<1 ng/mL) were observed in 297 individuals, the median was 0 ng/mL (IQR 0 - 0.33 ng/mL), while the highest level was 55.89 ng/mL. We found significantly higher sIFNAR2 levels among survivors than non-survivors (p=0.027) (**Supplementary Figure 2**). Four individuals exhibited high receptor levels, observed as outliers in the graph (>30 ng/mL). The clinical and demographic data were revised for each individual. However, we did not observe a striking similarity: two of them survived, three were females, age range 55-66 years, they were no smokers, mostly without the studied comorbidities, one was overweight, and three presented obesity, the days since symptoms onset vary 2-11 days, and all presented PaO_2_/FiO_2_ <200. We performed the analysis again, excluding the outliers, and the significant difference in the sIFNAR2 levels among groups remained (p=0.015, **Figure 1**). The subsequent analyses were carried out without the outliers (n=347)

In addition, higher sIFNAR2 levels were found among patients that did not use invasive mechanical ventilation when compared to those ventilated (0.05 ng/ml [0.00-0.82 ng/mL] vs. 0.00 ng/mL [0.00-0.15 ng/mL]). A Spearman’s correlation test showed a low correlation between sIFNAR2 levels and the length (days) of invasive mechanical ventilation (p=0.004, rho= -0.160). Nevertheless, this information should be cautiously considered since some patients requiring invasive mechanical ventilation did not accept the procedure, implying a possible bias in the study.

**Figure 2:**
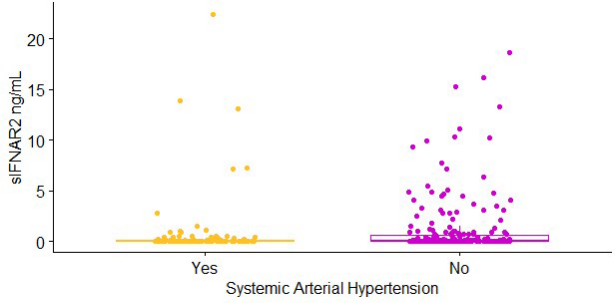
Soluble IFNAR2 (sIFNAR2) plasma levels of severe COVID-19 patients (n = 347) divided according to the comorbidity systemic arterial hypertension (SAH) (Yes: n = 121, yellow dots; No: n = 226, purple dots). sIFNAR2 level was evaluated by ELISA. Statistical comparison was performed using Mann-Whitney U Test, p < 0.01.

We evaluated if non-genetic factors influenced the sIFNAR2 values. Differences were observed when systemic arterial hypertension was considered (Mann-Whitney U Test, p=0.003) (**Figure 2**), and a weak correlation was found between sIFNAR2 levels and age or BMI (p<0.001, ρ=-0.253; p=0.012, ρ=0.135). Meanwhile, no differences in the receptor levels were observed according to sex, respiratory and ischemic heart diseases, or diabetes, although higher sIFNAR2 levels were observed in patients without the comorbidities (**Supplementary Figures 3**-**5**).

Moreover, the plasma levels of sIFNAR2 were compared according to the blood type as a previous report has suggested that cytokines’ levels are different for the O and A/B/AB individuals (20). We found a marginal difference in sIFNAR2 values according to the blood groups (p=0.048), but the difference was lost when the outliers were excluded (p=0.112) (**Supplementary Figure 6**).

Moreover, we evaluated whether the sIFNAR2 levels were influenced by the sampling time. We did not find a correlation between the sIFNAR2 levels and the sampling day after symptoms onset (p=0.857, ρ=0.010, Spearman’s correlation test), and no difference in the sampling day after symptoms onset was observed among the survivor and non-survivor groups (9 [6-9 days] vs. 8 [5-9 days], p=0.122, Mann-Whitney U test). Finally, we neither observed any influence of the *IFNAR2* rs2236757, rs1051393, rs3153, rs2834158, and rs2229207 on the sIFNAR2 plasma levels in individuals with severe COVID-19 (**Supplementary Figures 7**-**11**).

## Discussion

The dynamics of cytokines have been crucial in individuals’ progress with COVID-19. Variability in the cytokines and their receptors levels are related to the severity and clinical outcome of COVID-19. To the best of our knowledge, this is the first study reporting the plasma levels of sIFNAR2 in patients with COVID-19 and their association with the mortality risk of individuals with severe disease.

The association of *IFNAR2* locus with COVID-19 severity has been reported in different GWAS and multi-omic analyses (9, 10, 13, 14), as well as in a transcriptome-wide association study (21). In the present study, the *IFNAR2* rs2236757, rs2834158, rs3153, and rs1051393 were associated with mortality risk.

The rs2236757 was associated in a GWAS including individuals with critical illness in COVID-19 (9); herein, we also found an association with mortality in individuals with severe COVID-19. The departure from the Hardy-Weinberg equilibrium limits the magnitude of the finding, but, on the other hand, this probably highlights the relevance of the locus in the severity and mortality of the disease. Unfortunately, there is insufficient available data to compare the frequencies with other Mexican reports and drive additional conclusions.

The rs2236757, rs3153, and rs2834158 are intron variants previously explored in response to pegylated interferon-2a plus ribavirin to treat chronic hepatitis C virus infection (22). The frequencies of these *IFNAR2* variants present a relevant interethnic variability (23) that warrants further studies in different populations and elucidates the impact of these variants on the structure and/or function of the receptor IFN α/β. Nevertheless, the present report confirms the relevance of the *IFNAR2* locus in the severity and mortality of COVID-19.

The rs1051393 is a missense variant leading to a change of phenylalanine to valine in the 10th amino acid, and it is located in the signal peptide region affecting the IFNAR2 protein trafficking the membrane. This variant has been previously associated with chronic Hepatitis B virus infection, including 3,128 subjects of Han Chinese (24). According to their results, the authors suggested that the *IFNAR2* variants affect the receptor’s expression, limiting the antiviral effects of the IFN α/β. The rs1051393 has also been studied in colorectal cancer susceptibility and survival (25) and radiation-induced toxicity following the treatment of non-small cell lung cancer (26).

Although conclusions are controversial, several cytokines’ plasma levels have been related to COVID-19 severity and the clinical outcome. The IFN I and III levels have been related to COVID-19 susceptibility and severity (27, 28). Although the plasma levels of sIFNAR2 have not been previously reported, a reduced expression of *IFNAR2* was associated with COVID-19 severity (21). In agreement, we observed lower levels of the soluble receptor in the non-survivors group. Therefore, the relevance of the interferon pathway, mainly IFNAR2, in the COVID-19 severity has been evidenced at the genetic and transcription level and now with the amount of the soluble protein in plasma samples.

We observed extremely low plasma levels of the sIFNAR2 in most patients. The ELISA kit employed in this study presents a low limit detection (0.16 ng/mL), but the determination with lower quantification systems may be required. However, the decreased concentration of sIFNAR2 found in our study agrees with previous studies describing that the SARS-CoV-2 proteins inhibit the IFN-I pathway (29–31), resulting in a decline of IFN-α and -β among patients with COVID-19 (28, 32). Moreover, the higher sIFNAR2 levels observed among the survivor group compared to non-survivors match with the enhanced IFN antiviral activity due to the stability of the cytokine conferred by the sIFNAR2 at moderate concentrations (approximately 12 ng/mL) (33). This finding suggests that the sIFNAR2 could be implicated in the stability of the remaining IFN after infection with SARS-CoV-2.

Unfortunately, we could not assess the sIFNAR2 levels in uninfected individuals. However, a previous investigation reported sIFNAR2 levels in serum samples from healthy controls above those found in our study (median 134.3 ng/mL [IQR 76.10–179.21 ng/ml]) (34). In addition, this study reported the stability of the sIFNAR2 stored at -20°C and after four cycles of freezing/thawing, which shows the low risk of receptor degradation during the sample storage.

Regarding the blood group, we did not find significant differences in the sIFNAR2 plasma levels according to the ABO blood group of individuals with COVID-19, contrary to the previously reported for other cytokines and such as TNF-α, IFN-α, and several other cytokines and interleukins (20). Although higher receptor values were observed among individuals with A/B/AB groups compared to the O group, additional studies are required to clarify the relevance of the blood group in the prognosis of COVID-19.

The plasma levels of the receptor were also different considering the comorbidity of systemic arterial hypertension. The lower sIFNAR2 levels observed in individuals with hypertension could contribute to the critical and mortality risk of COVID-19; although, only a marginal p-value was observed for this variable in **Table 1** (p=0.06). In the scientific literature, only cases of pulmonary arterial hypertension related to IFN-β treatment have been reported (35, 36). Therefore, further studies could be required to clarify this difference in the sIFNAR2 levels according to the hypertension condition and if this is related to the severity of COVID-19.

The levels of several circulating cytokines have been found disturbed in COVID-19 and other infectious diseases, which is related to the disease severity and clinical outcome. The involvement of particular cytokines gives a clue about the pathophysiologic mechanisms involved in the diseases and the main immune pathways involved in the severity of the disease. Our findings highlight the relevance of the IFNAR2 pathway in the severe COVID-19, so this could be considered for the clinical management of the diseases or the therapeutic design.

Our study is not exempt from limitations. We could not recruit individuals with mild or moderate COVID-19 since the study center is a tertiary-care hospital; therefore, the relevance of IFNAR2 in less severe COVID-19 or asymptomatic individuals requires further investigation. In addition, the determination of sIFNAR2 plasma levels in healthy subjects, with evidence of no current or prior SARS-CoV-2 infection, would be interesting. Nevertheless, this report contributes to the severe COVID-19 insight and provides information for the design of further studies and the target of new and repurposed drugs.

## Data availability statement

The datasets presented in this study can be found in online repositories. The names of the repository/repositories and accession number(s) can be found below: https://www.ncbi.nlm.nih.gov/clinvar/, SCV002515911 - SCV002515915.

## Ethics statement

The studies involving human participants were reviewed and approved by Research Ethics Committee (C53-20). The patients/participants provided their written informed consent to participate in this study.

## Author contributions

RF-V and IF-G contributed to the conception and design of the study. IB-R, AA-D, HA-D, and JA-P organized the database. IB-R, MM, BB-B and RdJH-Z performed the clinical evaluation of patients. GP-R, KJN-Q and AC performed the statistical analysis. IF-G, IPG-G and AM-M wrote the first draft of the manuscript. AM-M, IAG-P, OZ-G and AV-A performed the genetic analysis. LC-G and RO-G performed the proteins’ determination. All authors contributed to manuscript revision and read and approved the submitted version.

## Acknowledgments

The authors acknowledge the support received from physicians and technicians from the clinical services at INER to confirm the study participants’ diagnosis and clinical care.

## Conflict of interest

The authors declare that the research was conducted without any commercial or financial relationships that could be construed as a potential conflict of interest.

## Publisher’s note

All claims expressed in this article are solely those of the authors and do not necessarily represent those of their affiliated organizations, or those of the publisher, the editors and the reviewers. Any product that may be evaluated in this article, or claim that may be made by its manufacturer, is not guaranteed or endorsed by the publisher.
