# Supplementary material for: IFNAR2 relevance in the clinical outcome of individuals with severe COVID-19
Source: Front Immunol. 2022 Jul 29;13:949413. doi: 10.3389/fimmu.2022.949413 (PMC9374460; doi:10.3389/fimmu.2022.949413)
Supplement: Supplementary file 1 [file DataSheet_1.docx]

Supplementary Material

# Supplementary Tables

**Supplementary Table 1. Recessive model analyses for *IFNAR2* genetic variants included in the study**

| *IFNAR2* variant | Genotypes | Non-survivors n=426 | Survivors  n=776 | p |
| --- | --- | --- | --- | --- |
| rs2834158 | CC  TC+ TT | 91 (0.214)  335 (0.786) | 136 (0.175)  640 (0.825) | 0.10 |
| rs2236757* | GG  AG+ AA | 102 (0.239)  324 (0.761) | 163 (0.210)  613 (0.790) | 0.23 |
| rs3153 | GG  AG+ AA | 92 (0.216)  334 (0.784) | 146 (0.188)  630 (0.012) | 0.24 |
| rs1051393 | TT  GT + GG | 92 (0.216)  334 (0.784) | 143 (0.184)  633 (0.816) | 0.18 |
| rs2229207 | CC  TC+ TT | 15 (0.035)  411 (0.965) | 28 (0.036)  748 (0.964) | 0.93 |

^*^ Departure from Hardy Weinberg Equilibrium p<0.01

# Supplementary Figures


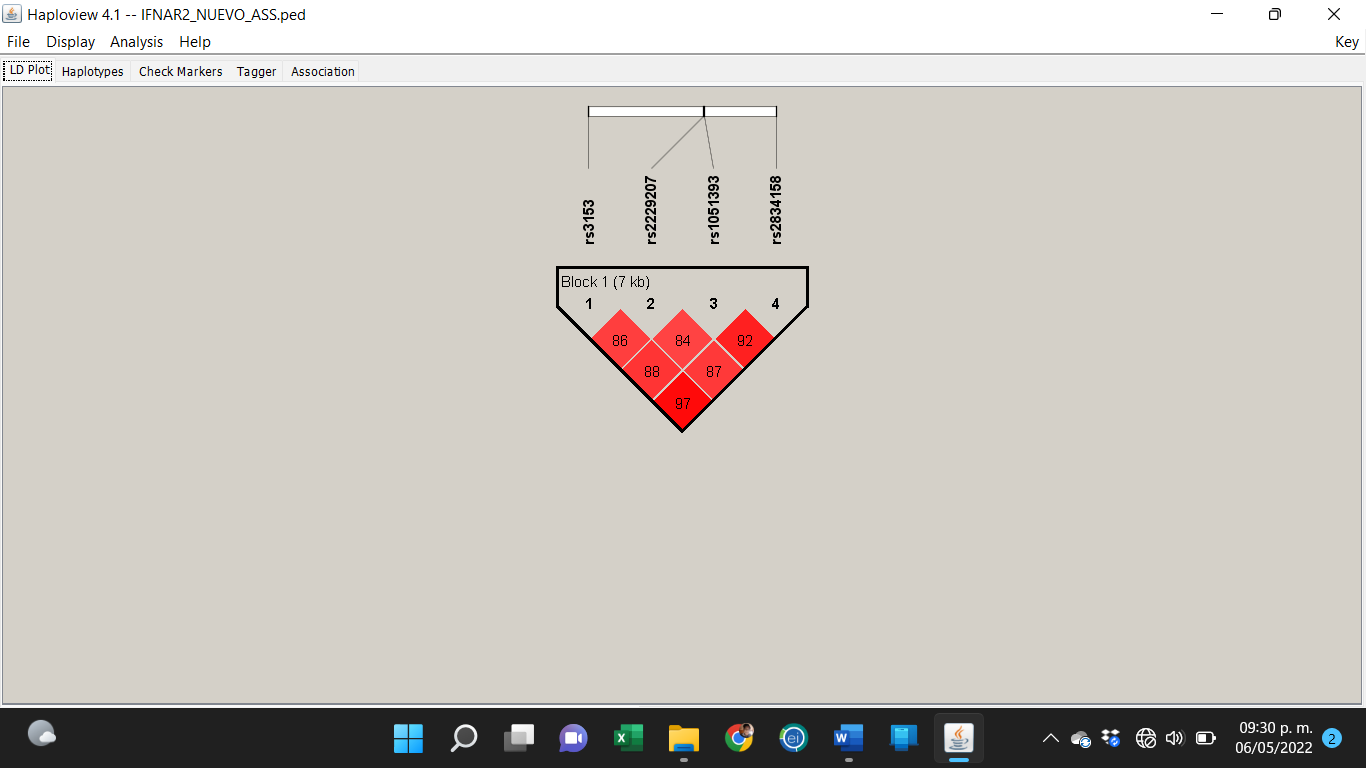

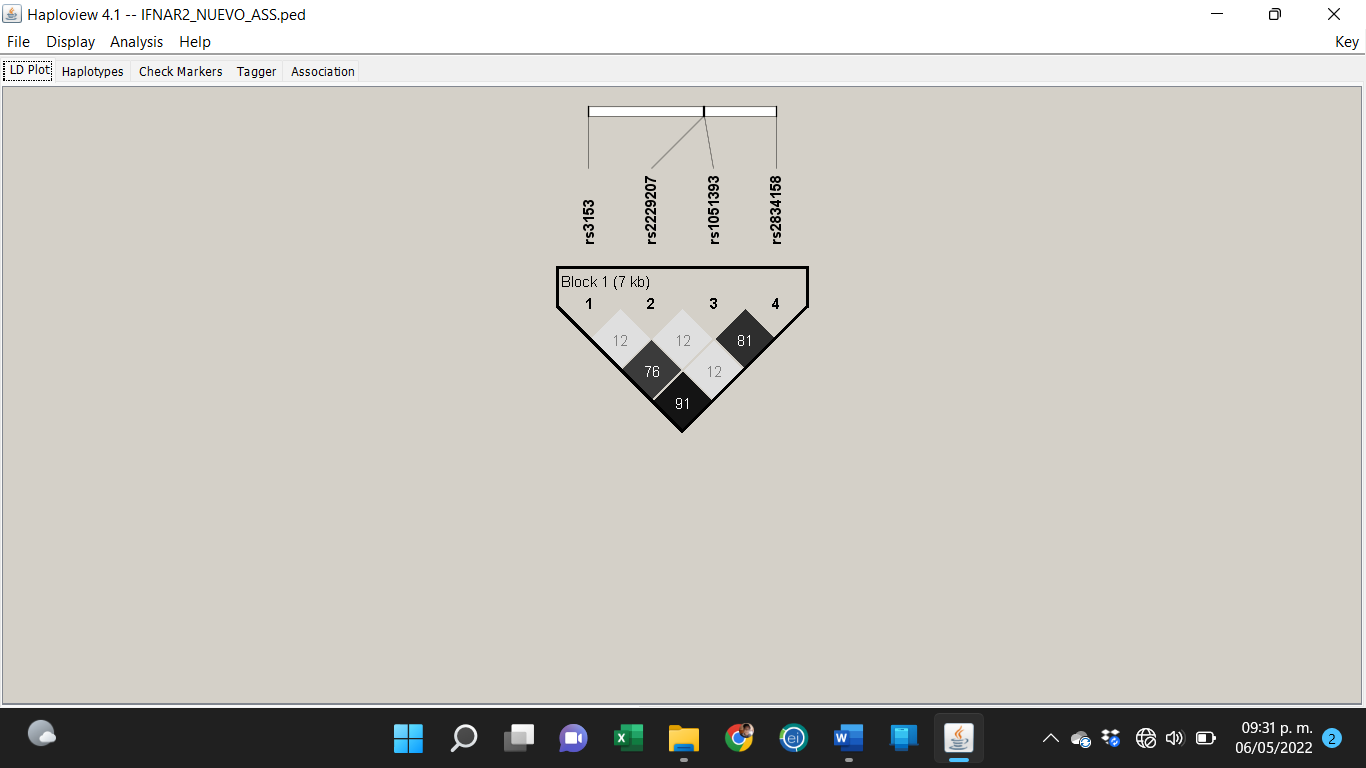


**(b)**

**(a)**

**Supplementary Figure 1. Linkage disequilibrium analysis for the IFNAR2 variants included in the study. rs2236757 was excluded due to the deviation to Hardy Weinberg equilibrium. (a) *D'* values; (b) *r^2^* values. A high color intensity is related to a strongest linkage disequilibrium among the genetic variants.**

**
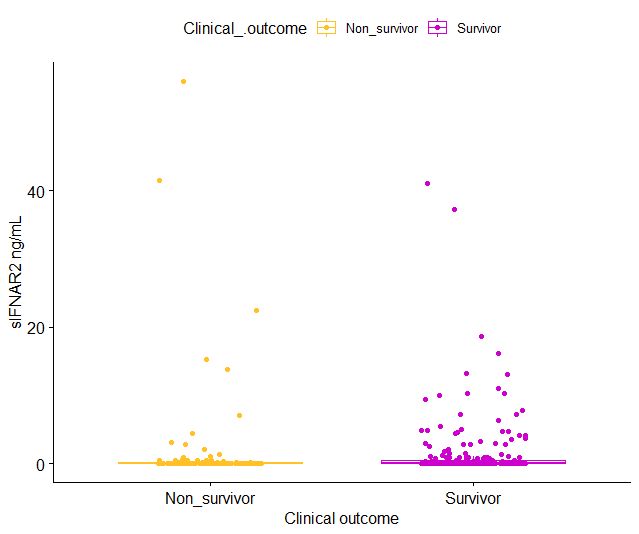
**

**Supplementary Figure 2. Soluble IFNAR2 (sIFNAR2) plasma levels of severe COVID-19 patients (n=351) divided into non-survivor (n=110, yellow dots) and survivor (n=241, purple dots).** sIFNAR2 level was evaluated by ELISA. Statistical comparison was performed using Mann-Whitney U Test, p<0.05.

**
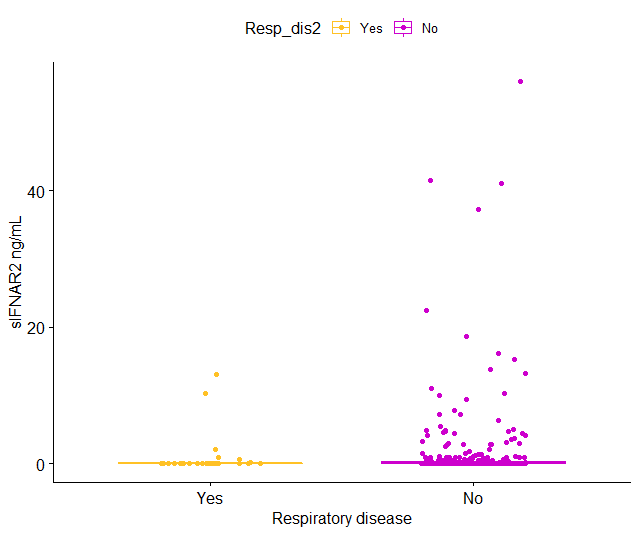
**

**Supplementary Figure 3. Soluble IFNAR2 (sIFNAR2) plasma levels of severe COVID-19 patients (n=351) divided according to the comorbidity chronic respiratory disease (Yes: n=26, yellow dots; No: n=325, purple dots).** sIFNAR2 level was evaluated by ELISA. Statistical comparison was performed using Mann-Whitney U Test, p>0.05.

**
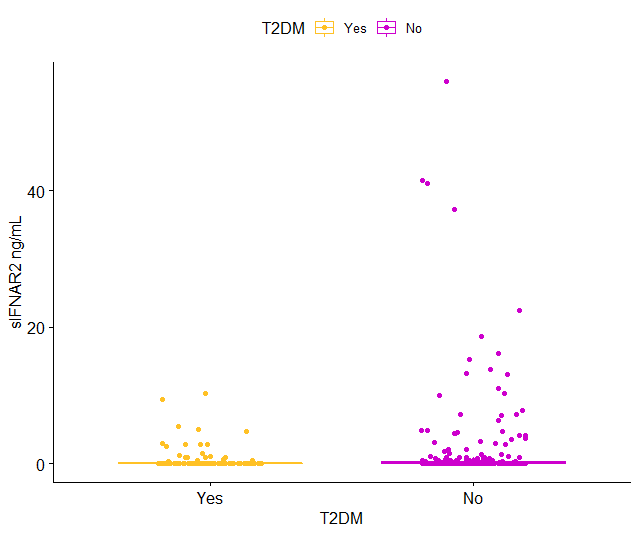
**

**Supplementary Figure 4. Soluble IFNAR2 (sIFNAR2) plasma levels of severe COVID-19 patients (n=351) divided according to the comorbidity type 2 diabetes mellitus (Yes: n=91, yellow dots; No: n=260, purple dots).** sIFNAR2 level was evaluated by ELISA. Statistical comparison was performed using Mann-Whitney U Test, p>0.05.

**
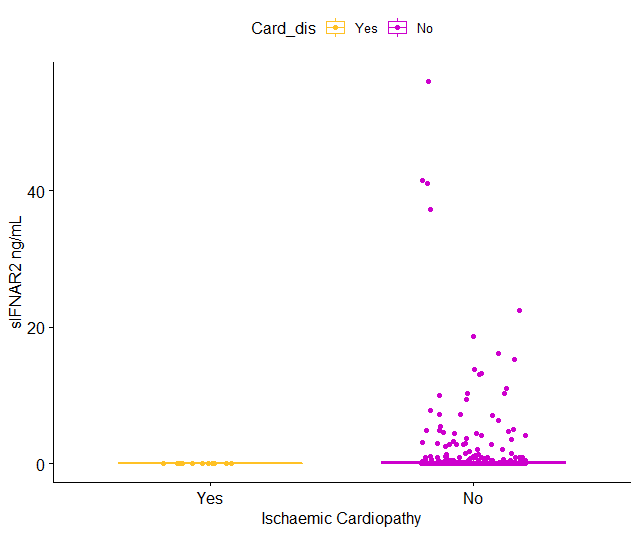
**

**Supplementary Figure 5. Soluble IFNAR2 (sIFNAR2) plasma levels of severe COVID-19 patients (n=351) divided according to the comorbidity type 2 ischemic heart disease (Yes: n=11, yellow dots; No: n=340, purple dots).** sIFNAR2 level was evaluated by ELISA. Statistical comparison was performed using Mann-Whitney U Test, p>0.05.

**
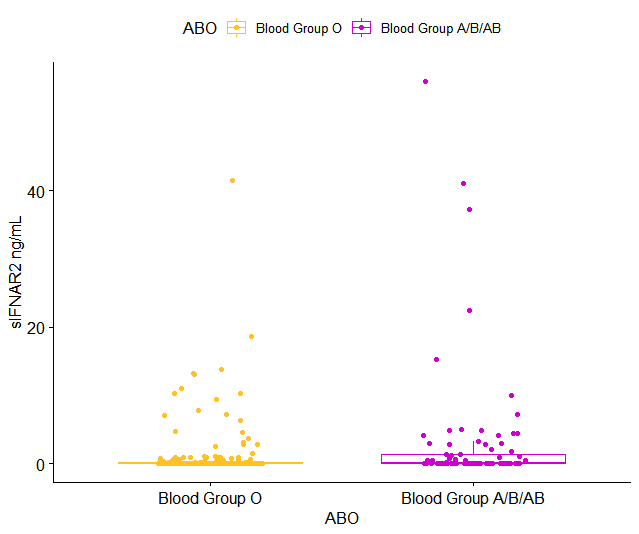
**
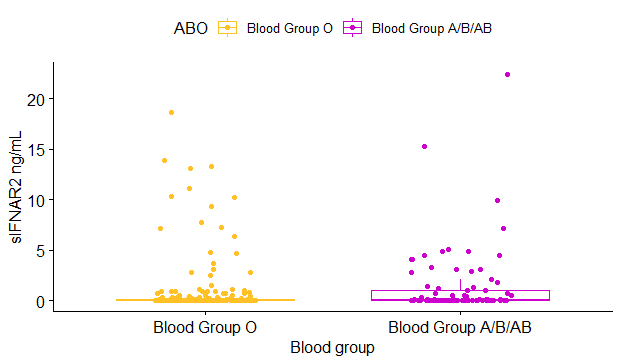


(B)

(A)

**Supplementary Figure 6. Soluble IFNAR2 (sIFNAR2) plasma levels of severe COVID-19 patients divided according to the blood group. (A) n=302, p=0.048 (Blood Group O: n=213, yellow dots; Blood Group A/B/AB: n=89, purple dots). (B) Excluding the outliers (sIFNAR2>30 ng/mL) n=298, p=0.112 ((Blood Group O: n=212, yellow dots; Blood Group A/B/AB: n=86, purple dots).** sIFNAR2 level was evaluated by ELISA. Statistical comparison was performed using Mann-Whitney U Test.

**
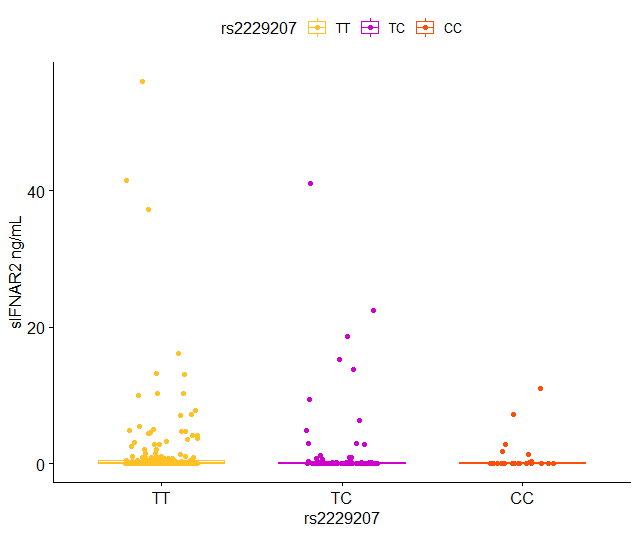
**

**Supplementary Figure 7. Soluble IFNAR2 (sIFNAR2) plasma levels of severe COVID-19 patients (n=351) divided according to the genotypes of *IFNAR2* rs2229207 (TT n= 231, TC n= 94, CC n=26).** sIFNAR2 level was evaluated by ELISA. Statistical comparison was performed using Mann-Whitney U Test, p>0.05.

**
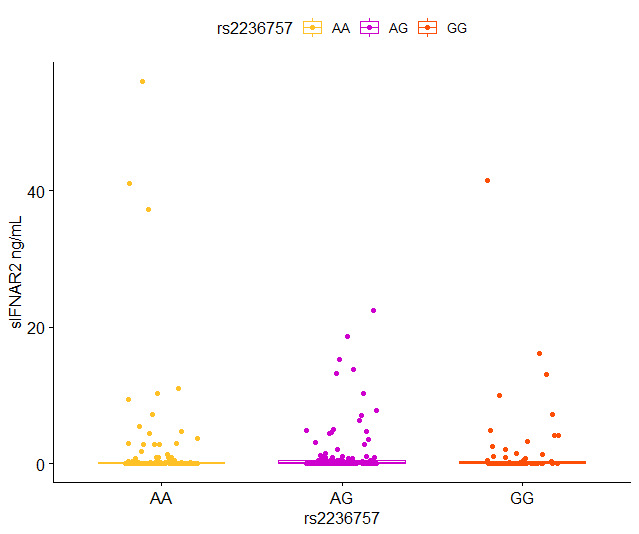
**

**Supplementary Figure 8. Soluble IFNAR2 (sIFNAR2) plasma levels of severe COVID-19 patients (n=351) divided according to the genotypes of *IFNAR2* rs2236757 (AA n=131, AG n=144, GG n=76).** sIFNAR2 level was evaluated by ELISA. Statistical comparison was performed using Mann-Whitney U Test, p>0.05.

**
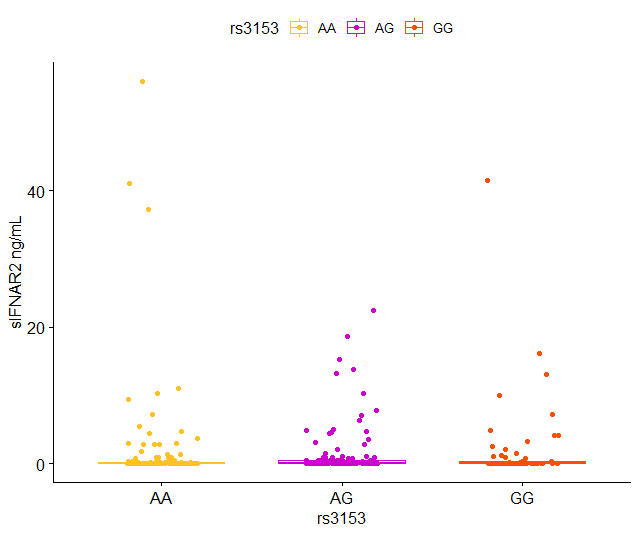
**

**Supplementary Figure 9. Soluble IFNAR2 (sIFNAR2) plasma levels of severe COVID-19 patients (n=351) divided according to the genotypes of *IFNAR2* rs3153 (AA n=132, AG n=147, GG n=72).** sIFNAR2 level was evaluated by ELISA. Statistical comparison was performed using Mann-Whitney U Test, p>0.05.

**
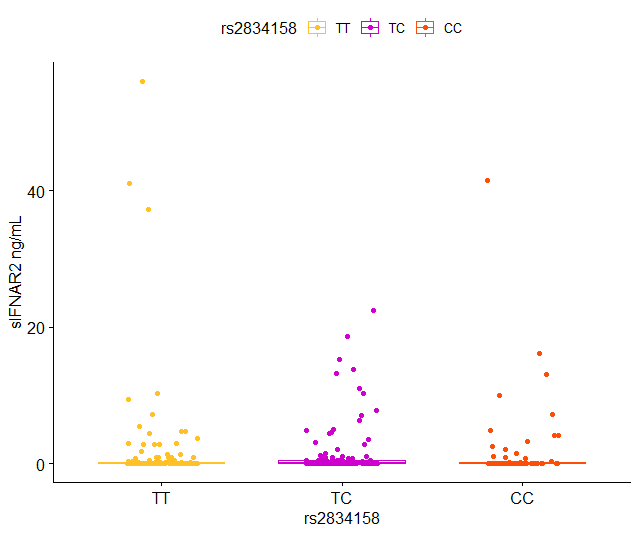
**

**Supplementary Figure 10. Soluble IFNAR2 (sIFNAR2) plasma levels of severe COVID-19 patients (n=351) divided according to the genotypes of *IFNAR2* rs2834158 (TT n=134, TC n=151; CC n=66).** sIFNAR2 level was evaluated by ELISA. Statistical comparison was performed using Mann-Whitney U Test, p>0.05.

**
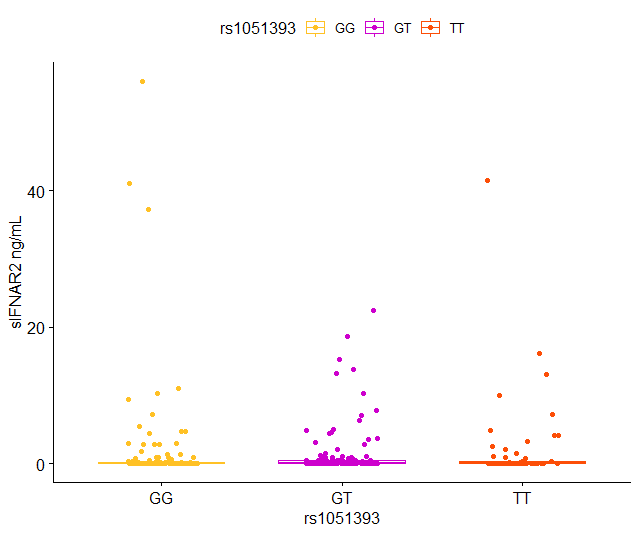
**

**Supplementary Figure 11. Soluble IFNAR2 (sIFNAR2) plasma levels of severe COVID-19 patients (n=351) divided according to the genotypes of *IFNAR2* rs1051393 (GG n=129, GT n=155, TT n=67).** sIFNAR2 level was evaluated by ELISA. Statistical comparison was performed using Mann-Whitney U Test, p>0.05.
